# An Analysis of a Compact Label-Free Guiding-Wave Biosensor Based on a Semiconductor-Clad Dielectric Strip Waveguide

**DOI:** 10.3390/s20123368

**Published:** 2020-06-14

**Authors:** Carlos Angulo Barrios

**Affiliations:** 1 Institute for Optoelectronic Systems and Microtechnology (ISOM), ETSI Telecomunicación, Universidad Politécnica de Madrid, Ciudad Universitaria s/n, 28040 Madrid, Spain; carlos.angulo.barrios@upm.es; 2 Department of Photonics and Bioengineering (TFB), ETSI Telecomunicación, Universidad Politécnica de Madrid, Ciudad Universitaria s/n, 28040 Madrid, Spain

**Keywords:** optical biosensor, photonic modeling, waveguide, integrated optics

## Abstract

In this paper, a compact, integrated, semiconductor-clad strip waveguide label-free biosensor is proposed and analyzed. The device is based on CMOS-compatible materials such as amorphous-Si and silicon oxynitride. The optical sensor performance has been modeled by a three-dimensional beam propagation method. The simulations indicate that a 20-μm-long device can exhibit a surface limit of detection of 3 ng/cm^2^ for avidin molecules in aqueous solution. The sensor performance compares well to those displayed by other photonic biosensors with much larger footprints. The fabrication tolerances have been also studied in order to analyze the feasibility of the practical implementation of the biosensor.

## 1. Introduction

Integrated optics-based biosensors offer a number of remarkable features such as their small size, high-scale integration, high sensitivity, robustness and potential for multiplexed detection that make them ideal for lab-on-chip integration [1,2,3]. These compact devices are particularly well-suited for label-free detection schemes since they are able to measure small refractive index changes produced by the recognition of unlabeled analytes [4]. The use of Si-based materials provides additional and important advantages, like the possibility of employing highly developed fabrication techniques based on the CMOS technology and integration with advanced readout electronics on the same chip. Thus, a variety of Si-based integrated photonic biosensors have been reported in the literature, including Mach–Zehnder [5,6,7,8] and Young [9,10] interferometers, bimodal waveguides [11], microcavities [12,13,14,15] and photonic crystals [16,17].

Semiconductor and metal-clad optical waveguides allow for the modulation of the properties of propagating light due to coupling between the lossless modes of the dielectric waveguide and the lossy optical modes supported by the thin cladding layer [18,19,20,21]. This coupling depends on the thickness and refractive index of the cladding layer, as well as on the refractive index of the surrounding medium, which makes this type of guided-wave structures suitable for refractometric (bio)sensing [22,23,24,25,26,27,28]. Compared to metal-clad configurations, the use of a semiconductor layer cladding allows for the use of both transverse electric (TE) (the electric field has no component in the direction of propagation) and transverse magnetic (TM) (the magnetic field has no component in the direction of propagation) polarization modes [18,19] and offers the possibility of obtaining higher refractive index sensitivities [25]. However, despite a semiconductor-clad waveguide is highly amenable to be integrated on planar substrates, scarce work has been devoted to study this prospect for, for example, lab-on-chip biosensing microsystems; the vast majority of semiconductor-clad waveguide biosensors have been demonstrated using optical fibers [25,26,27,28].

In this work, an integrated, semiconductor-clad strip waveguide biosensor based on CMOS-compatible materials is proposed and analyzed. The device optical performance, sensitivity to both bulk refractive index and adlayer (biofilm) thickness, and tolerance to dimensional and material parameter variations have been studied through three-dimensional numerical modeling. Simulations indicate that the proposed device shows good sensing characteristics to be used as a compact photonic label-free biosensor, and provide important information concerning its actual implementation.

## 2. Device Configuration and Modeling

**Figure 1:**
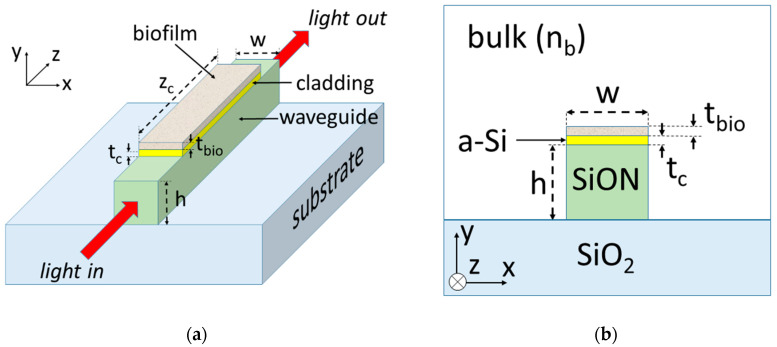
Perspective (**a**) and cross-sectional (**b**) schematics of a semiconductor-clad dielectric strip waveguide biosensor. The cladding, waveguide and substrate are assumed to be amorphous-Si (a-Si), silicon oxynitride (SiON) and SiO_2_, respectively. The biofilm consists of a layer of biomolecules such as proteins.

Figure 1a,b show a perspective and cross-section schematics, respectively, of the proposed guided-wave optical biosensor. It consists of a thin semiconductor layer (cladding) deposited on the top surface of a lossless dielectric strip waveguide on a silicon dioxide (SiO_2_) substrate. The width of both the cladding layer and the strip waveguide is w = 1 μm. The semiconductor cladding layer thickness equals t_c_ and the height of the strip waveguide is h = 1 μm. The length of the semiconductor cladding layer is denoted as z_c_. The cladding and waveguide materials are assumed to be amorphous silicon (a-Si) and silicon oxynitride (SiON), respectively. The refractive indices of a-Si, SiON and SiO_2_ at a free-space wavelength of 632.8 nm (operation wavelength) have been considered to be n_Si_ = 4.1 − j0.21 [29], n_wg_ = 1.52 [30] and n_sub_ = 1.46, respectively. The upper cover region (bulk) has a refractive index of n_b_. Both the upper cover and substrate regions are assumed to be semi-infinite in extent. For the biosensing analysis, a uniform protein film (biofilm) of thickness t_bio_, width w, length z_c_, and refractive index n_bio_ = 1.41 [31] has been assumed to be adhered on the semiconductor cladding layer in an aqueous medium (n_b_ = 1.33).

The bio-sensitive area of the optical device is the top surface of the semiconductor cladding layer, where biomolecule receptors (e.g., antibodies) can be immobilized or adsorbed (biofilm in Figure 1a). Light, at an operating wavelength of 632.8 nm, is injected at the input port of the waveguide and the optical power exiting the output port is used as the sensor response. Analyte recognition by the immobilized bioreceptors produces a change in the biofilm thickness and, therefore, in the local refractive index over the cladding layer, which affects the optical power coupling between the lossy semiconductor layer and the lossless dielectric waveguide. This is displayed as an optical power variation at the output of the waveguide.

A three-dimensional beam propagation method (BPM) [32] was used for the calculation of the modal-field profile and optical power propagating along the waveguide (*z*-axis). The computational grid and step sizes along x, y and z were Δx = 50 nm, Δy = 0.5 nm and Δz = 50 nm, respectively. Transparent boundary conditions in the computational domain were used in the simulations.

## 3. Results

**Figure 2:**
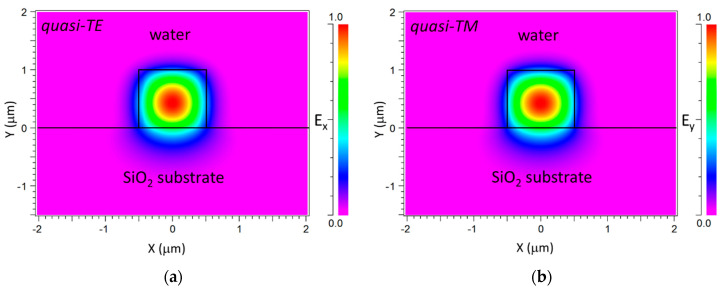
Transverse electric field profiles of the quasi-transverse electric (TE) (**a**) and quasi-transverse magnetic (TM) (**b**) fundamental optical modes in a bare dielectric (SiON) waveguide (t_c_ = t_bio_ = 0) at λ = 632.8 nm in an aqueous medium. E_x_ (a) and E_y_ (b) are the electric field components along the *x*-axis and *y*-axis, respectively.

The bare dielectric strip waveguide configuration (that is, t_c_ = t_bio_ = 0) exhibits single mode operation for both quasi-TE and quasi-TM polarization modes when the upper cover is assumed to be water (n_b_ = 1.33). Figure 2 shows the corresponding optical mode-field profiles for the quasi-TE (Figure 2a) and quasi-TM (Figure 2b) fundamental modes. The calculated effective refractive indices were 1.4811 and 1.4818, respectively. Thus, the launched (input) fields used in the BPM simulations of the device for t_c_ ≥ 0 were the fundamental modes of the bare dielectric waveguide for the corresponding polarization.

**Figure 3:**
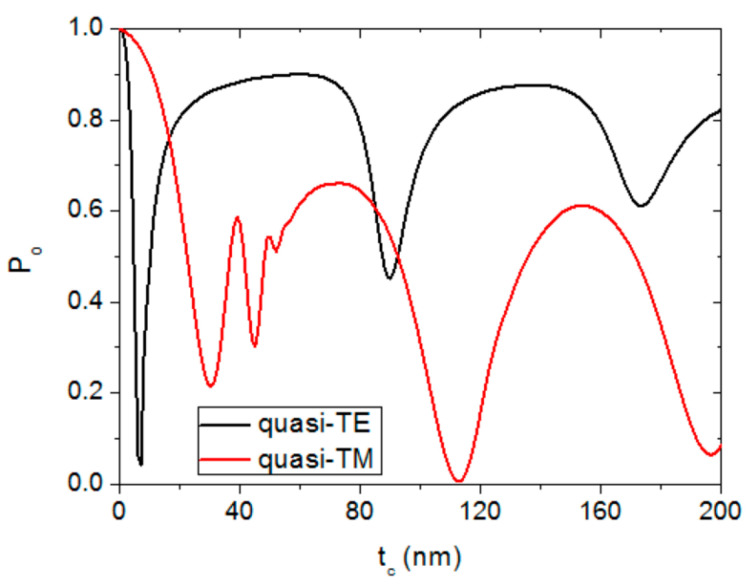
Normalized power at the output (P_o_) of a 20-μm-long a-Si-clad SiON strip waveguide as a function of the semiconductor thickness value for quasi-TE (black line) and quasi-TM (red line) polarizations. n_b_ = 1.33 and t_bio_ = 0.

Figure 3 plots the calculated output power (P_o_), normalized to the input power, of the studied guided-wave device as a function of the semiconductor layer thickness t_c_ for quasi-TE (black line) and quasi-TM (red line) polarizations for n_b_ = 1.33, z_c_ = 20 μm and t_bio_ = 0. Quasi-TE and quasi-TM polarizations exhibit different behaviors, indicating different guide-cladding interactions. This is a consequence of the different sets of boundary conditions for each polarization [18,19]. Several transmission dips are observed for both polarizations in Figure 3. These output power transmission minima (attenuation maxima) arise from the periodic coupling effect between the lossless mode of the dielectric waveguide and the lossy mode of the thin semiconductor cladding layer [18,19].

**Figure 4:**
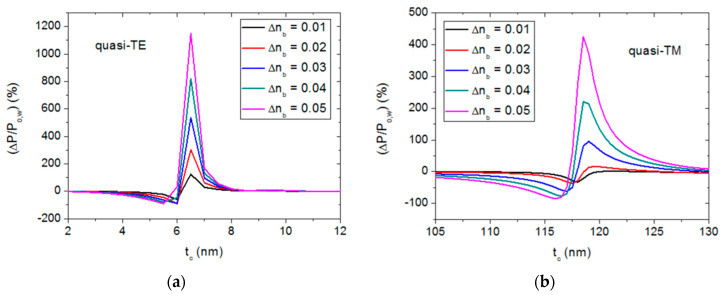
Relative power variation in a 20-μm-long semiconductor-clad dielectric strip waveguide as a function of the semiconductor thickness (t_c_) for different bulk refractive index variations (Δn_b_) with respect to n_b_ = 1.33, for quasi-TE (**a**) and quasi-TM (**b**) polarizations.

BPM simulations revealed that variations in the bulk refractive index n_b_ leads to shifts of the transmission dips along the t_c_ axis, implying that the output power is sensitive to the bulk refractive index for particular t_c_ values. Figure 4 shows the relative output power variation (ΔP/P_o,w_), where P_o,w_ is the normalized output power for n_b_ = 1.33, for quasi-TE (Figure 4a) and quasi-TM (Figure 4b) polarizations, and different n_b_ variations (Δn_b_) in the t_c_ ranges where (ΔP/P_o,w_) is largest. It is seen that, for quasi-TE mode, the maximum relative power variation occurs for t_c_ = 6.5 nm for all considered Δn_b_ values, whereas, for quasi-TM polarization, the corresponding t_c_ value depends on the bulk refractive index change, and varies between 121 nm (Δn_b_ = 0.01) and 118.5 nm (Δn_b_ = 0.05). It should be noted that the peaks in Figure 4a have larger amplitudes and are sharper than the corresponding curves in Figure 4b, indicating that the quasi-TE operation mode is more sensitive to both n_b_ and t_c_ variations than the quasi-TM polarization mode.

**Figure 5:**
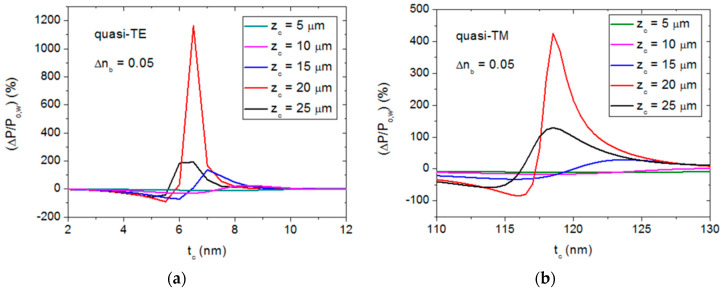
Relative power variation in the considered semiconductor-clad dielectric strip waveguide configuration as a function of the semiconductor thickness (t_c_) for a bulk refractive index variation (Δn_b_) of 0.05 (1.38–1.33) and different semiconductor layer lengths (z_c_) for quasi-TE (**a**) and quasi-TM (**b**) polarizations.

Figure 5 shows the effect of the semiconductor layer length, z_c_, on the relative output power variation for a particular bulk refractive index variation (Δn_b_ = 0.05) for quasi-TE (Figure 5a) and quasi-TM (Figure 5b) polarizations. In both cases, the maximum relative power values occur for z_c_ = 20 μm. Therefore, hereafter the length of the semiconductor layer will be assumed to be 20 μm for both quasi-TE and quasi-TM operation modes, and, according to Figure 4, the thickness of the semiconductor cladding layer will be 6.5 nm and 118 nm for quasi-TE and quasi-TM polarizations, respectively.

### 3.1. Bulk Sensitivity

Intensity-based refractive index optical sensors typically use as a figure-of-merit the relative power (or intensity) bulk sensitivity defined as [33,34,35]:

(1) $$S_{I,b}=\frac{1}{P_{o}}\left(\frac{dP_{o}}{dn_{b}}\right),$$

**Figure 6:**
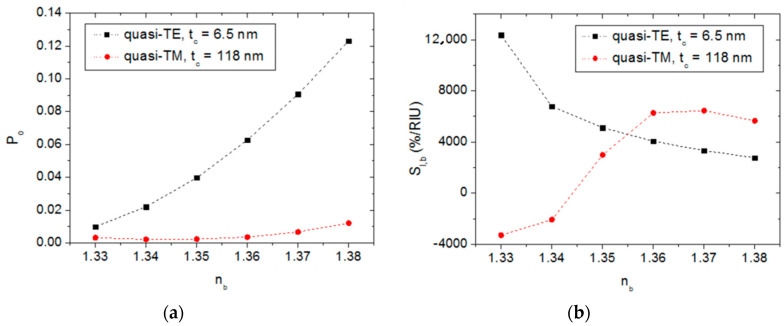
Sensor response (**a**) and bulk sensitivity (**b**) of the studied semiconductor-clad dielectric strip waveguide as a function of the bulk refractive index for quasi-TE (black square dots) and quasi-TM (red circular dots) polarizations. The semiconductor layer thickness values are t_c_ = 6.5 nm and t_c_ = 118 nm for quasi-TE and quasi-TM operations, respectively. Data points are connected by dashed lines for the sake of clarity.

Figure 6a illustrates the calculated sensor response for quasi-TE polarization (black square dots). It is seen that P_o_ increases monotonically in a slightly non-linear fashion as n_b_ increases. This leads to a positive and increasing value of the sensor response derivative. However, P_o_ increases faster with n_b_ than the derivative does, and counteracts the gain effect of the latter on S_I,b_. The resulting consequence is a bulk sensitivity value that depends on the n_b_ value as shown in Figure 6b (black square dots). For the calculation of S_I,b_ from the data of Figure 6a, the derivative of P_o_ at the *i*-th point was computed as (P_o(i+1)_ − P_o(i−1)_)/(n_b(i+1)_ − n_b(i−1)_), and the derivatives for the first (i = 1) and last (i = 6) points were calculated as (P_o2_ − P_o1_)/(n_b2_ − n_b1_) and (P_o6_ − P_o5_)/(n_b6_ − n_b5_), respectively. It is seen, in Figure 6b, that S_I,b_ decreases monotonically as n_b_ increases, and exhibits a maximum value of 12,372%/RIU for n_b_ = 1.33. Quasi-TE operation is therefore particularly suitable for refractive index sensing of diluted aqueous solutions. Assuming a minimum detectable intensity difference of 1%, a bulk refractive index resolution of 8 × 10^−5^ RIU [ = 1%/12,372 (%/RIU)] should be attainable. For the sake of comparison, Table 1 shows the intensity bulk sensitivity, limit of detection (LOD), product LOD × L_sens_, where L_sens_ is the length of the sensing region, and footprint of relevant Si-based planar refractive index optical sensors reported in the literature. It is seen that the device analyzed in this work exhibits better sensitivity than those based on free-space optical interrogation [34,35,36]. Optical sensors based on integrated-optics [8,11] present smaller LODs but at the expense of a long sensing region. In fact, the product LOD × L_sens_, which is a convenient figure of merit for these type of sensors, of the studied device is similar or even better (smaller) than those of the other integrated guided-wave devices, while offering a footprint five orders of magnitude smaller. The latter is an important advantage for large-scale integration on a single chip.

The sensor response for quasi-TM polarization is also shown in Figure 6a (red circular dots). In this case, the response is clearly non-linear and non-monotonic. This leads to a magnitude and sign n_b_-dependent behaviour of the bulk sensitivity, as shown in Figure 6b (red circular dots). The maximum sensitivity, 6456%/RIU, is obtained for n_b_ = 1.37. Thus, the quasi-TM operation of the sensor could be useful for testing liquids other than aqueous solutions, such as, for example, some organic solvents.

### 3.2. Biofilm Sensing

Like the bulk sensitivity, the relative power (or intensity) thickness sensitivity S_I,t_ can be defined as:

(2) $$S_{I,t}=\frac{1}{P_{o}}\left(\frac{dP_{o}}{dt_{bio}}\right),$$

**Figure 7:**
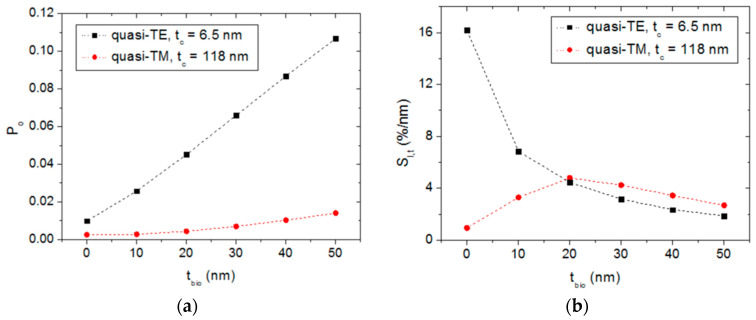
Sensor response (**a**) and thickness sensitivity (**b**) of the studied semiconductor-clad dielectric strip waveguide as a function of the biofilm thickness for quasi-TE (black square dots) and quasi-TM (red circular dots) polarizations. The semiconductor layer thickness values are t_c_ = 6.5 nm and t_c_ = 118 nm for quasi-TE and quasi-TM operations, respectively. Data points are connected by dashed lines for the sake of clarity.

Figure 7a shows the sensor response as a function of the biofilm thickness for quasi-TE and quasi-TM polarizations. The corresponding thickness sensitivities are plotted in Figure 7b. For quasi-TE polarization (black square dots) a nearly linear response is obtained in the entire t_bio_ range. The highest quasi-TE sensitivity, S_I,t_ = 16.2%/nm, is achieved for t_bio_ = 0 nm. This thickness sensitivity implies that for a minimum detectable intensity difference of 1%, the smallest detectable biofilm thickness would be 0.06 nm. For a typical protein such as avidin, modeled as a sphere of diameter 5 nm and molecular weight of 50 kD, the surface density of a monolayer of proteins would be 254 ng/cm^2^ [37]. Thus, assuming that the sensor signal is proportional to the surface coverage, the minimum detection limit would be 3 ng/cm^2^. This value is larger than high-performance plasmonic [37] and porous Si [38] biosensors based on free-space and wavelength interrogation. However, it compares well to the performance of other intensity-based integrated optics Si-based biosensors as shown in Table 2. In particular, the product LOD × L_sens_ exhibited by the analyzed device is less than those biosensors based on integrated Mach–Zehnder [6,8] and Young interferometers [10], and its footprint is several orders of magnitude smaller. Figure 7b also shows that the sensitivity for quasi-TE polarization decreases as the biofilm thickness increases, which is mainly a consequence of the increment of P_o_ with t_bio_. Quasi-TE operation is therefore particularly well suited for detecting very thin layers of proteins deposited directly on the semiconductor cladding layer.

The response for quasi-TM polarization (red circular dots) shows no variation for t_bio_ up to 10 nm and a slight linear variation for larger t_bio_ values. The quasi-TM thickness sensitivity lies between 0.9%/nm and 4.8%/nm, exhibiting similar values to those of quasi-TE polarization for t_bio_ greater than 20 nm. The low sensitivity for t_bio_ = 0 (0.9%/nm) indicates that, for quasi-TM operation, the sensing device should contain a pre-deposited (or immobilized) biofilm of thickness equal or greater than 10 nm in order to function at a higher sensitivity operating point. Such a biofilm could be made up by one or several monolayers of biomolecule receptors such as antibodies.

### 3.3. Material and Dimension Tolerance

From a fabrication perspective, geometrical and material parameter deviations from the target values can always occur. An analysis of the sensitivity of the device performance to these deviations allows critical fabrication parameters to be identified, assisting the technologists in determining or developing proper processing techniques.

**Figure 8:**
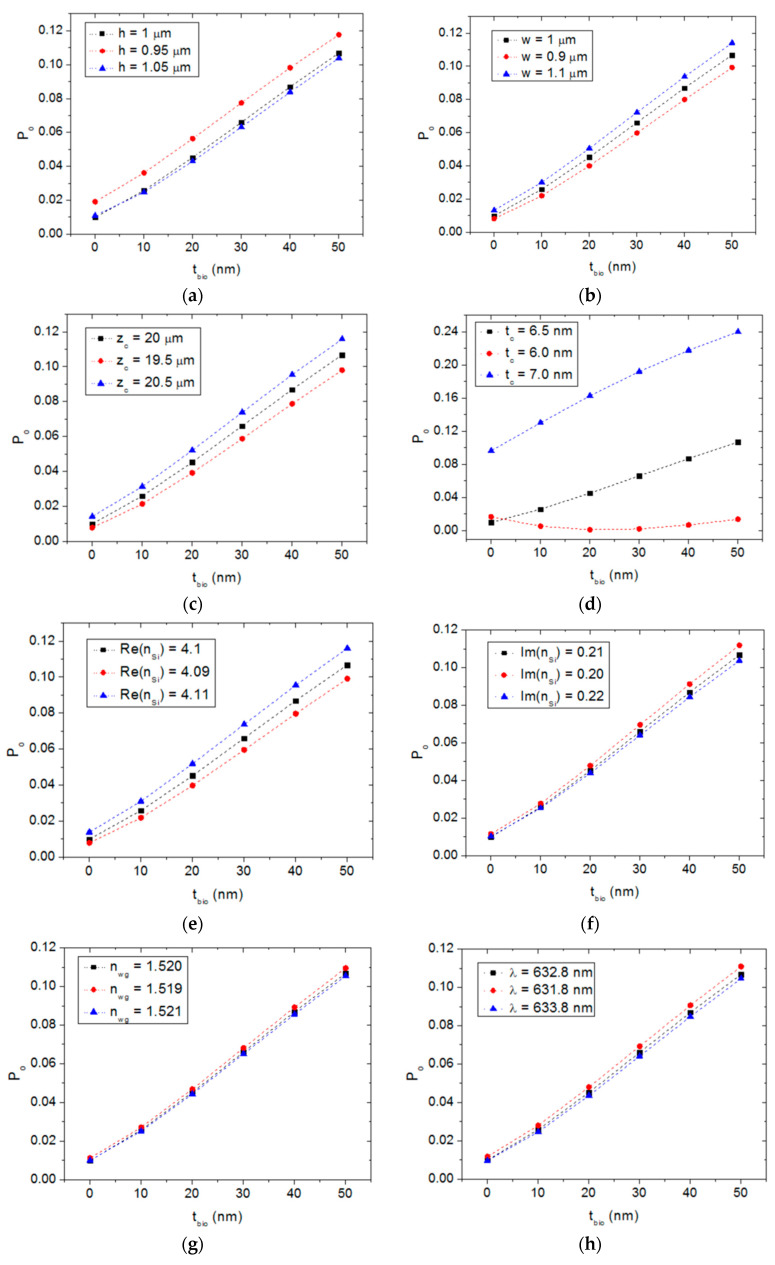
Normalized power at the output of a target a-Si-clad SiON strip waveguide as a function of the biofilm thickness for different dimensional and material parameter variations: (**a**) waveguide height variation, (**b**) waveguide width variation, (**c**) semiconductor cladding length variation, (**d**) semiconductor cladding thickness variation, (**e**) variation in the real part of the semiconductor cladding refractive index, (**f**) variation in the imaginary part of the semiconductor cladding refractive index, (**g**) SiON waveguide refractive index variation, and (**h**) operation wavelength variation.

Since the quasi-TE polarization mode exhibits both a larger response variation and surface sensitivity than those obtained for the quasi-TM mode, only the former operation mode has been considered in this tolerance analysis. Thus, for a target device characterized by: h = w = 1 μm, z_c_ = 20 μm, t_c_ = 6.5 nm, Re(n_Si_) = 4.1, Im(n_Si_) = 0.21, n_wg_ = 1.52 and λ = 632.8 nm, Figure 8 shows the sensor response (device output power as a function of the biofilm thickness) for individual variations in h (Figure 8a), w (Figure 8b), z_c_ (Figure 8c), t_c_ (Figure 8d), Re(n_Si_) (Figure 8e), Im(n_Si_) (Figure 8f), and n_wg_ (Figure 8g).

These figures reveal that the most critical fabrication parameter is the thickness of the a-Si cladding layer. Acceptable tolerances are obtained for the rest of the considered parameters. From the sensor operation point of view, it is also relevant to analyze the effect of deviations in the operating wavelength on the sensor performance. Wavelength variation can be originated by, for example, thermal fluctuations affecting the light source (laser device). Figure 8h shows that a variation of ± 1 nm in the operating wavelength has a negligible effect on the sensor response.

## 4. Discussion

The integrated device analyzed in this work is based on a single channel. There is no need for the Y-shape splitters used in integrated Mach–Zehnder and Young interferometer biosensors, which significantly increase the footprint and are complicated to design and fabricate. Besides, the sensor response is obtained by monitoring the waveguide output optical intensity (or power). Intensity-based read-out schemes essentially require a fixed wavelength source (typically a single wavelength laser device) and a photodetector, simplifying the overall sensing system; interrogation schemes based on wavelength shift measurement require more complex and costly equipment such as a tunable laser source or a spectrum analyzer.

The choice of silicon oxynitride for the waveguide material has been motivated by the purpose of obtaining single mode operation at the operating wavelength using micron-sized cross-sectional waveguide dimensions and Si-based materials. Waveguides with micron-sized cross sections facilitate both direct optical coupling (butt coupling) and fabrication compared to submicrometer- size waveguides. SiON can be deposited by conventional chemical vapor deposition (CVD) techniques [6,10,30] and, depending on composition, its refractive index can be varied between that of silicon dioxide and that of silicon nitride, offering high potential and flexibility for optical waveguide design.

**Figure 9:**
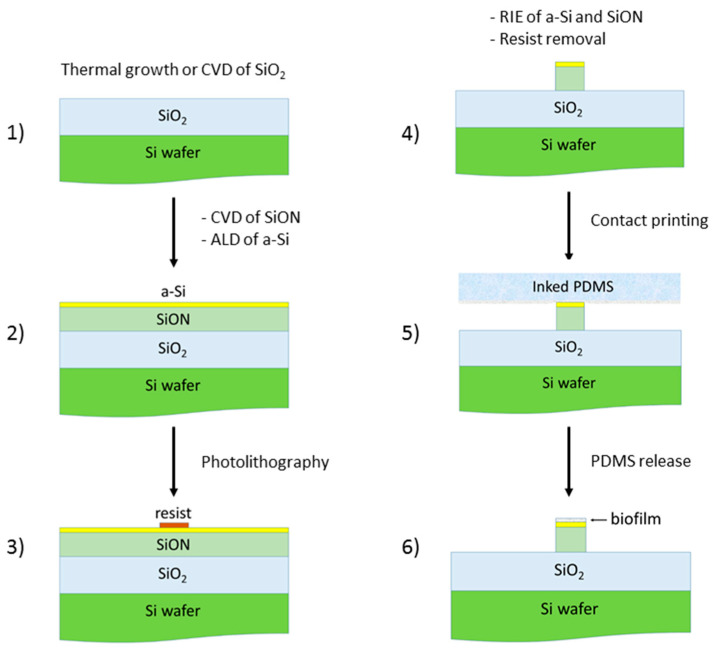
Schematic diagram of the main processing steps involved in the fabrication of the proposed integrated optical biosensor. Chemical vapor deposition (CVD); reactive ion etching (RIE); atomic layer deposition (ALD); polydimethylsiloxane (PDMS).

The micrometer dimensions of the dielectric waveguide (1 μm × 1 μm × 20 μm) allow for its fabrication using standard methods from the microelectronics industry such as CVD, photolithography and reactive ion etching (RIE). There is no need for complex and expensive nanolithographic processes. Figure 9 illustrates a schematic description of the main processing steps involved in the fabrication of the studied sensor on a Si wafer. The simulation results have indicated that the most challenging fabrication issue is the deposition of the a-Si layer, whose thickness should be controlled to the monolayer level (approximately, 0.5 nm). This task can be carried out by atomic layer deposition (ALD), which is a well-known and mature technology that allows for the deposition of ultrathin films of dielectric and semiconductor materials, such as Si [39,40,41], with precisely controlled thickness at the atomic scale and high uniformity over large areas. Note that, unlike the difficulties of nanopatterning lateral features, very high-quality thin films can be deposited with sub-nanometer control on a substrate. Finally, the device can be converted into a biosensor by immobilizing biomolecules on the surface of the a-Si cladding film, which could be achieved by contact printing with PDMS (polydimethylsiloxane) inked with proper biomolecules [42,43].

To conclude, the performance of the modeled integrated optical biosensor, particularly for quasi-TE polarization, compares well to state-of-the-art Si-based planar optical biosensors based on intensity interrogation while presenting a significantly smaller footprint. The analyzed device is therefore a promising microcomponent for use in lab-on-a-chip biosensing platforms based on Si-based integrated optics.

## Figures and Tables

**Table 1:** Si-based planar refractive index (RI) optical sensors based on intensity interrogation. Refractive index unit (RIU); Mach–Zehnder (M–Z); limit of detection (LOD); length of the sensing region (L_sens_); not applicable (N/A).

RI Sensor Configuration	Sensitivity (%/RIU)	LOD (RIU)	LOD × L_sens_ (RIU mm)	Footprint (μm^2^)
Metasurface [34]	4450	(2 × 10^−4^) ^b^	N/A ^c^	N/A ^c^
Grating/waveguide [35]	1306	(7.6 × 10^−4^) ^b^	N/A ^c^	N/A ^c^
1-D grating [36]	1700	(5 × 10^−4^) ^b^	N/A ^c^	N/A ^c^
M–Z interferometer [8]		5.4 × 10^−6^	3.8 × 10^−5^	3.4 × 10^6^
Bimodal waveguide [11]		2.5 × 10^−7^	3.7 × 10^−6^	4.5 × 10^6^
*This work*	12,372 ^a^	(8 × 10^−5^) ^b^	1.6 × 10^−6^	20

^a^ Maximum value. Sensitivity depends on the bulk refractive index. ^b^ For a change in relative output intensity of 1%. ^c^ Free-space interrogation.

**Table 2:** Si-based integrated optical biosensors based on intensity interrogation. Mach–Zehnder (M–Z); limit of detection (LOD); length of the sensing region (L_sens_).

Biosensor Configuration	LOD (ng/cm^2^)	LOD × L_sens_ (ng cm^−2^ mm)	Footprint (μm^2^)
M–Z interferometer [6]	2	24	3 × 10^6^
M–Z interferometer [8]	0.22	1.54	3.4 × 10^6^
Young interferometer [10]	0.075	0.9	1.8 × 10^7^
*This work*	3 ^a^	0.06	20

^a^ Minimum value (sensitivity depends on the adlayer thickness), for a change in relative output intensity of 1%.

## References

[1] Zinoviev K., Carrascosa L.G., del Río J.S., Sepúlveda B., Domínguez C., Lechuga L.M. (2008). Silicon photonic biosensors for lab-on-a-chip applications. Adv. Opt. Technol..

[2] Washburn A.L., Bailey R.C. (2011). Photonics-on-a-chip: Recent advances in integrated waveguides as enabling detection elements for real-world, lab-on-a-chip biosensing applications. Analyst.

[3] Gavela A.F., García D.G., Ramirez J.C., Lechuga L.M. (2016). Last advances in silicon-based optical biosensors. Sensors.

[4] Luan E., Shoman H., Ratner D.M., Cheung K.C., Chrostowski L. (2018). Silicon Photonic Biosensors Using Label-Free Detection. Sensors.

[5] Heideman R.G., Kooyman R.P.H., Greve J. (1993). Performance of a highly sensitive optical waveguide Mach-Zehnder interferometer immunosensor. Sens. Actuators B.

[6] Weisser M., Tovar G., Mittler-Neher S., Knoll W., Brosinger F., Freimuth H., Lacher M., Ehrfeld W. (1999). Specific bio-recognition reactions observed with an integrated Mach–Zehnder interferometer. Biosens. Bioelectron..

[7] Schipper E., Brugman A., Dominguez C., Lechuga L., Kooyman R., Greve J. (1997). The realization of an integrated Mach–Zehnder waveguide immunosensor in silicon technology. Sens. Actuators B Chem..

[8] Liu Q., Tu X., Kim K.W., Kee J.S., Shin Y., Han K., Yoon Y., Lo G., Park M.K. (2013). Highly sensitive Mach-Zehnder interferometer biosensor based on silicon nitride slot waveguide. Sens. Actuators B Chem..

[9] Brandenburg A. (1997). Differential refractometry by an integrated-optical Young interferometer. Sens. Actuators B Chem..

[10] Brandenburg A., Krauter R., Künzel C., Stefan M., Schulte H. (2000). Interferometric sensor for detection of surface-bound bioreactions. Appl. Opt..

[11] Zinoviev K.E., González-Guerrero A.B., Domínguez C., Lechuga L.M. (2011). Integrated bimodal waveguide interferometric biosensor for label-free analysis. J. Lightwave Technol..

[12] Krioukov E., Klunder D.J., Driessen A., Greve J., Otto C. (2002). Sensor based on an integrated optical microcavity. Opt. Lett..

[13] Yalçin A., Popat K.C., Aldridge J.C., Desai T.A., Hryniewicz J., Chbouki N., Little B.E., King O., Van V., Chu S. (2006). Optical sensing of biomolecules using microring resonators. IEEE J. Sel. Top. Quantum Electron..

[14] De Vos K., Bartolozzi I., Schacht E., Bienstman P., Baets R. (2007). Silicon-on-Insulator microring resonator for sensitive and label-free biosensing. Opt. Express.

[15] Barrios C.A., Bañuls M.J., Gonzalez-Pedro V., Gylfason K.B., Sánchez B., Griol A., Maquieira A., Sohlström H., Holgado M., Casquel R. (2008). Label-free optical biosensing with slot waveguides. Opt. Lett..

[16] Lee M., Fauchet P.M. (2007). Two-dimensional silicon photonic crystal based biosensing platform for protein detection. Opt. Express.

[17] Scullion M.G., Falco A.D., Krauss T.F. (2011). Slotted photonic crystal cavities with integrated microfluidics for biosensing applications. Biosens. Bioelectron..

[18] Carson R.F. (1985). Periodic Coupling in Semiconductor-Clad Dielectric Optical Guided-Wave Devices. Ph.D. Thesis.

[19] Carson R.F., Batchman T.E. (1990). Multimode phenomena in semiconductor-clad dielectric optical waveguide structures. Appl. Opt..

[20] Kaminow I.P., Mammel W.L., Weber H.P. (1974). Metal-Clad Optical Waveguides: Analytical and Experimental Study. Appl. Opt..

[21] Rashliegh S.C. (1975). Planar Metal-Clad Dielectric Optical Waveguides. Ph.D. Thesis.

[22] Slavik R., Homola J., Ctyroky J., Brynda E. (2001). Novel spectral fiber optic sensor based on surface plasmon resonance. Sens. Actuators B.

[23] Zourob M., Goddard N.J. (2005). Metal clad leaky waveguides for chemical and biosensing applications. Biosens. Bioelectron..

[24] Skivesen N., Horvath R., Thinggaard S., Larsen N.B., Pedersen H.C. (2007). Deep-probe metal-clad waveguide biosensors. Biosens. Bioelectron..

[25] Andreev A., Pantchev B., Danesh P., Zafirova B., Karakoleva E., Vlaikova E., Alipieva E. (2005). A refractometric sensor using index-sensitive mode resonance between single-mode fiber and thin film amorphous silicon waveguide. Sens. Actuators B.

[26] Socorro A.B., Corres J.M., Del Villar I., Arregui F.J., Matias I.R. (2012). Fiber-optic biosensor based on lossy mode resonances. Sens. Actuators B.

[27] Paliwal N., John J. (2015). Lossy Mode Resonance (LMR) Based Fiber Optic Sensors: A Review. IEEE Sens. J..

[28] Del Villar I., Arregui F.J., Zamarreño C.R., Corres J.M., Bariain C., Goicoechea J., Elosua C., Hernaez M., Rivero P.J., Socorro A.B. (2017). Optical sensors based on lossy-mode resonances. Sens. Actuators B.

[29] Pierce D.T., Spicer W.E. (1972). Electronic Structure of Amorphous Si from Photoemission and Optical Studies. Phys. Rev. B.

[30] Bossi D.E., Hammer J.M., Shaw J.M. (1987). Optical properties of silicon oxynitride dielectric waveguides. Appl. Opt..

[31] Vorös J. (2004). The density and refractive index of adsorbing protein layers. Biophys. J..

[32] Rsoft Photonic Device Tools. https://www.synopsys.com/photonic-solutions/rsoft-photonic-device-tools.html.

[33] Perera C., Vernon K., Cheng E., Sathian J., Jaatinen E., Davis T. (2016). Highly compact refractive index sensor based on stripe waveguides for lab-on-a-chip sensing applications. Beilstein J. Nanotechnol..

[34] Rodionov S.A., Remnev M.A., Klimov V.V. (2019). Refractive index sensor based on all-dielectric gradient metasurface. Sens. Bio-Sens. Res..

[35] Takashima Y., Kusaba K., Haraguchi M., Naoi Y. (2019). Highly sensitive refractive index sensor using dual resonance in subwavelength grating/waveguide with normally incident optical geometry. IEEE Sens. J..

[36] Shakoor A., Grande M., Grant J., Cumming D.R.S. (2017). One dimensional silicon nitride grating refractive index sensor suitable for integration with CMOS detectors. IEEE Photonics J..

[37] Dahlin A.B. (2012). Plasmonic Sensors: An Integrated View of Refractometric Detection.

[38] Lin V.S.-Y., Motesharei K., Dancil K.-P.S., Sailor M.J., Ghadiri M.R. (1997). A porous silicon-based optical interferometric biosensor. Science.

[39] Imai S., Iizuka T., Sugiura O., Matsumura M. (1993). Atomic layer epitaxy of Si using atomic H. Thin Solid Films.

[40] Hasunuma E., Sugahara S., Hoshino S., Imai S., Ikeda K., Matsumura M. (1998). Gas-phase-reaction-controlled atomic-layer-epitaxy of silicon. J. Vac. Sci. Technol. A.

[41] Ikeda K., Yanase J., Sugahara S., Matsumura M. (2001). Atomic-Layer-Epitaxy of Si. J. Korean Phys. Soc..

[42] Bernard A., Renault J.P., Michel B., Bosshard H.R., Delamarche E. (2000). Microcontact printing of proteins. Adv. Mater..

[43] LaGraff J.R., Chu-LaGraff Q. (2006). Scanning force microscopy and fluorescence microscopy of microcontact printed antibodies and antibody fragments. Langmuir.

